# Modelling population-level impact to inform target product profiles for childhood malaria vaccines

**DOI:** 10.1186/s12916-018-1095-6

**Published:** 2018-07-13

**Authors:** Alexandra B. Hogan, Peter Winskill, Robert Verity, Jamie T. Griffin, Azra C. Ghani

**Affiliations:** 1 Department of Infectious Disease Epidemiology, MRC Centre for Global Infectious Disease Analysis, Imperial College London, School of Public Health, St Mary’s Campus, Norfolk Place, London, W2 1PG UK; 2 School of Mathematical Sciences, Queen Mary University London, Mile End Road, London, E1 4NS UK

**Keywords:** RTS,S/AS01, Target product profile, *Plasmodium falciparum*, Malaria, Efficacy, Second-generation malaria vaccine

## Abstract

**Background:**

The RTS,S/AS01 vaccine for *Plasmodium falciparum* malaria demonstrated moderate efficacy in 5–17-month-old children in phase 3 trials, and from 2018, the vaccine will be evaluated through a large-scale pilot implementation program. Work is ongoing to optimise this vaccine, with higher efficacy for a different schedule demonstrated in a phase 2a challenge study. The objective of our study was to investigate the population-level impact of a modified RTS,S/AS01 schedule and dose amount in order to inform the target product profile for a second-generation malaria vaccine.

**Methods:**

We used a mathematical modelling approach as the basis for our study. We simulated the changing anti-circumsporozoite antibody titre following vaccination and related the titre to vaccine efficacy. We then implemented this efficacy profile within an individual-based model of malaria transmission. We compared initial efficacy, duration and dose timing, and evaluated the potential public health impact of a modified vaccine in children aged 5–17 months, measuring clinical cases averted in children younger than 5 years.

**Results:**

In the first decade of delivery, initial efficacy was associated with a higher reduction in childhood clinical cases compared to vaccine duration. This effect was more pronounced in high transmission settings and was due to the efficacy benefit occurring in younger ages where disease burden is highest. However, the low initial efficacy and long duration schedule averted more cases across all age cohorts if a longer time horizon was considered. We observed an age-shifting effect due to the changing immunological profile in higher transmission settings, in scenarios where initial efficacy was higher, and the fourth dose administered earlier.

**Conclusions:**

Our findings indicate that, for an imperfect childhood malaria vaccine with suboptimal efficacy, it may be advantageous to prioritise initial efficacy over duration. We predict that a modified vaccine could outperform the current RTS,S/AS01, although fourth dose timing will affect the age group that derives the greatest benefit. Further, the outcome measure and timeframe over which a vaccine is assessed are important when prioritising vaccine elements. This study provides insight into the most important characteristics of a malaria vaccine for at-risk groups and shows how distinct vaccine properties translate to public health outcomes. These findings may be used to prioritise target product profile elements for second-generation childhood malaria vaccines.

**Electronic supplementary material:**

The online version of this article (10.1186/s12916-018-1095-6) contains supplementary material, which is available to authorized users.

## Background

The success of modern vaccines has greatly reduced the global burden of infectious diseases, particularly for childhood infections [1, 2]. However, in sub-Saharan Africa, the childhood burden of malaria remains significant, even with the widespread use of vector control interventions and effective treatment, which have greatly reduced morbidity and mortality. As vaccines are being introduced for more complex diseases, the development of a vaccine for malaria has become a key global health priority. The first strategic goal in the World Health Organization’s Malaria Vaccine Technology Roadmap is the development of malaria vaccines with protective efficacy against clinical malaria of at least 75% over 2 years, for administration to appropriate at-risk groups in malaria-endemic areas, with a booster dose administered no more frequently than annually [3].

The most advanced vaccine is currently the RTS,S/AS01 vaccine for *Plasmodium falciparum* malaria. The phase 3 trial of RTS,S/AS01 was conducted over the period 2009–2014, in two target age groups and for three- and four-dose schedules. In infants aged 6–12 weeks at enrolment who received four doses of the trial vaccine, efficacy was 27.8% (21.7–33.4 95% CI) over a 32-month follow-up period. In 5–17-month-old children who received four doses, efficacy against clinical malaria was 43.9% (39.7–47.8 95% CI) over the same time period [4]. The RTS,S/AS01 vaccine is unlikely to be pursued as a viable vaccine for infants, due to the low observed efficacy. However, RTS,S/AS01 will now be evaluated through a large-scale pilot implementation program in 5–17-month-old children in three sub-Saharan Africa settings: Ghana, Kenya and Malawi [5].

Work is ongoing to improve the efficacy of the RTS,S/AS01 vaccine, and recent evidence has indicated that varying the timing and amount of the fourth dose could lead to greater efficacy and improved public health outcomes [6, 7]. In an RTS,S/AS01 challenge study of healthy adults, with a fractional third dose and fractional booster, efficacy against clinical disease was 86.7% (66.8–94.6 95% CI) at the first challenge (3 weeks after the third dose) and 43% (− 9 to 70 95% CI) at the second challenge (8 months after the first challenge). In the group that received a fractional booster (fourth dose) 8 months after the first challenge, efficacy was 90% (36–98 95% CI) at the second challenge. The immunological reason for this difference is not fully understood, although it may in part be due to improved affinity of the antibodies [7].

Target product profiles (TPPs) have traditionally been used by industry to guide vaccine and drug development, by setting preferred criteria for product safety, indication, efficacy and cost-effectiveness. However, there is increasing focus on using TPPs as more adaptable, broader tools that capture the full public health value of a drug or vaccine, to help a wider range of stakeholders, such as policy-makers, design and evaluate vaccine formulations [8]. For diseases with complex epidemiological features, such as malaria, mathematical modelling can be particularly useful for informing the public health impact components of TPPs [9].

In this study, we used mathematical models of malaria transmission and vaccine efficacy to predict the impact of childhood vaccination with a modified RTS,S/AS01 vaccine and to inform TPPs for second-generation vaccines, focussing on the elements of initial efficacy, duration of protection, dosing schedules and coverage [10–13]. We determined the relative importance of initial vaccine efficacy versus duration of protection in a range of *P. falciparum* prevalence settings, in terms of clinical cases averted in children younger than 5 years, for the first decade following vaccine introduction. We also considered how the timing and efficacy of the fourth dose are likely to change the public health impact. Finally, we explored options for realistic vaccine efficacy profiles for a modified RTS,S/AS01 vaccine, in line with the results of a recent study by Regules et al. [6], and used these profiles to estimate the impact of an enhanced vaccine in different transmission settings.

## Methods

### Modelling approach

Our modelling approach comprised two steps. First, we simulated the changing anti-circumsporozoite (anti-CSP) antibody titre following vaccination, and we then used a mathematical model relating antibody titre to efficacy against infection to establish the corresponding efficacy profile. Second, assuming the vaccine was delivered to young children, we implemented this efficacy profile within an individual-based model of malaria transmission, as has been described previously [12].

White et al. used data from African infants and children to analyse the determinants of immunogenicity after inoculation with the RTS,S/AS01 malaria vaccine and to develop a model relating these determinants to vaccine efficacy [13]. There were two components to the model: (1) a biphasic exponential function that simulated the changing antibody titre over time after the third dose of the vaccine and (2) a Hill function that captured the relationship between antibody titre and vaccine efficacy. We used the same biphasic exponential function to describe the time-dependent anti-CSP antibody titre resulting from inoculation with a malaria vaccine. This function simulated the initial increase, and then decay, of antibody titres induced by the combined short-lived and long-lived B-cell responses to vaccination. We used the fitted parameters described by White et al., Table 3, for the 5–17-month age category [13]. For the peak observed antibody titre, we applied the median of the 11 site values in the phase 3 RTS,S/AS01 trial, which was 621 EU/mL [13].

**Fig. 1:**
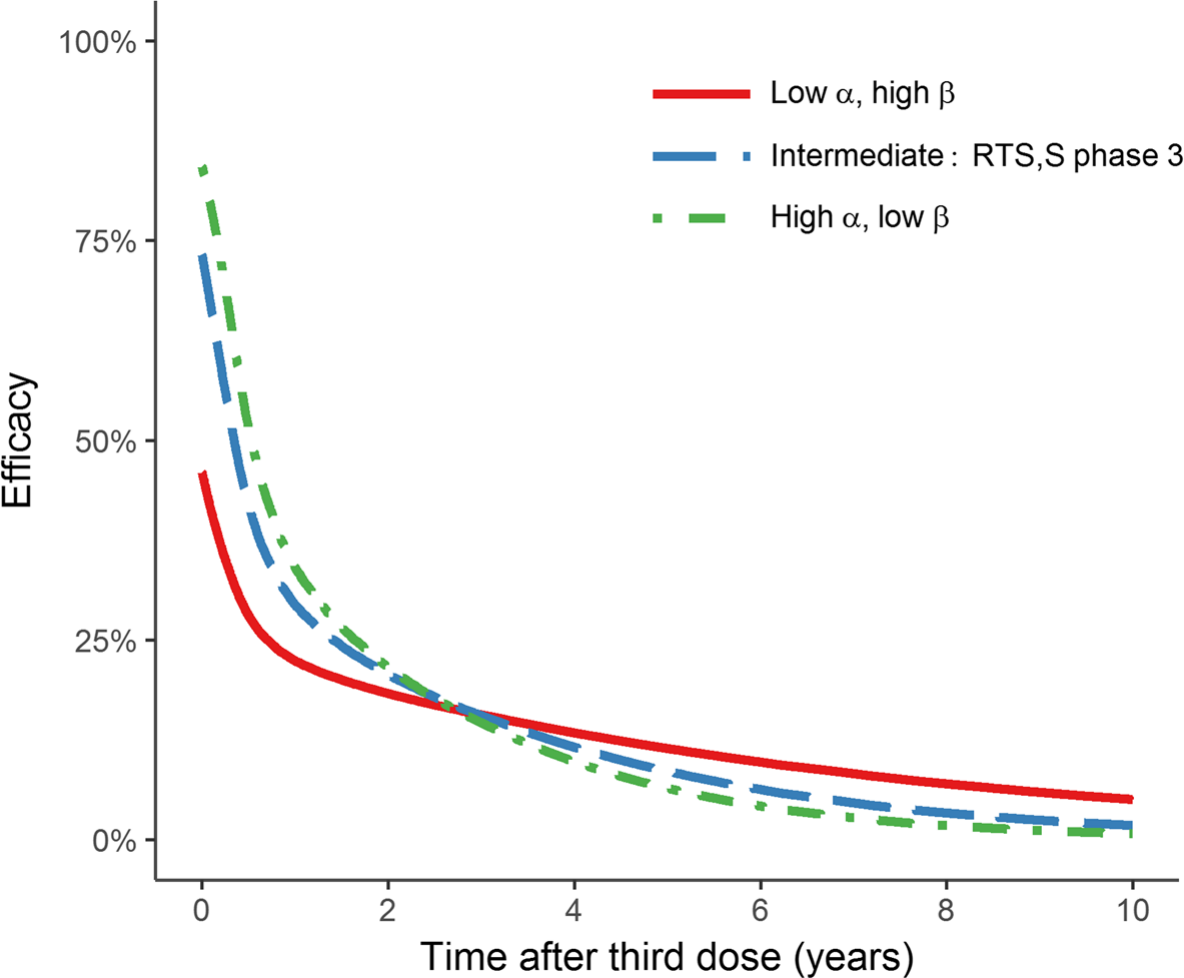
Profiles for three points on the α-β curve, where the area under the efficacy-time curve is held constant. *Red solid line* α = 0.40, β = 615.06, *V*_max_ = 0.93 (lower initial efficacy, longer duration). *Blue dashed line* α = 0.74, β = 99.20, *V*_max_ = 0.93 (RTS,S/AS01 phase 3 trial modelled parameters). *Green dash-dotted line* α = 1.00, β = 60.56, *V*_max_ = 0.93 (higher initial efficacy, shorter duration). The corresponding antibody titre curve for the intermediate scenario is shown in Additional file 1: Figure S2, and the role of the shape parameter α and scale parameter β is illustrated in Additional file 1: Figure S10

The Hill function used to simulate vaccine efficacy is:

$$ V(t)={V}_{max}\left(1-\frac{1}{1+{\left(\frac{CS(t)}{\beta}\right)}^{\alpha }}\right), $$

where *CS*(*t*) represents the modelled anti-CSP antibody titre (EU/mL) over time *t*, *V*_max_ represents the maximum efficacy against infection, α represents the shape parameter of the dose-response curve and β represents the scale parameter of the dose-response curve. In simple terms, α relates to the initial efficacy of the vaccine and β relates to the duration of protection (Fig. 1). Varying α and β allows us to model different antibody avidities for a given titre, resulting in different vaccine efficacy profiles (Additional file 1: Figure S10).

We used this formulation within a previously published mathematical transmission model for *P. falciparum* malaria transmission. The model pairs an individual-based model of the human transmission process with a stochastic compartmental model that captures mosquito biology. In the model, susceptible individuals are bitten by infectious mosquitoes at a rate driven by mosquito density and infectivity. Different disease states, treatment and temporary prophylaxis following a treated infection are incorporated, as are exposure-driven immunity and passive maternally derived immunity in the first 6 months of life. The individual-based transmission model has been previously validated using data on the relationship between the entomological inoculation rate and parasite prevalence, clinical disease, severe disease and deaths from multiple sites [14, 15]. Full details of the transmission model are available elsewhere [10, 11], with the key parameters summarised in Table 1.

**Table 1:** Summary of malaria transmission model parameters

Category	Settings
Prevalence settings	5–50% in 5% increments. Four distinct transmission settings, with P*f*PR_2–10_ at start year set to 5%, 15%, 30% and 45%
Simulation size	30,000–65,000, depending on prevalence level
Seasonality	Perennial, with Fourier coefficients for the perennial setting as in Walker et al. [22]
Treatment	40% coverage of artemisinin-based combination therapy (ACT) treatment with 95% efficacy
Vaccine coverage	Default value of 80% coverage for the first three vaccine doses, with fourth dose coverage at 80% of the coverage for the first three doses in line with Penny et al. [12]. Varied in the range 50–90% in 5% increments
Vaccine dose timing	First three RTS,S/AS01 vaccine doses administered at 5, 6.5 and 8 months of age. Fourth dose administered at 26 months (default) and varied in the range 16–30 months

*Pf*PR_2–10_ represents *P. falciparum* parasite prevalence in 2–10-year-old children

We ran the simulations for generalised settings that were characteristic of malaria-endemic regions in Africa. The starting *P. falciparum* prevalence for 2–10-year-old individuals, denoted as *Pf*PR_2–10_, was in the range 5 to 50%, and we selected four initial prevalence settings for comparison. This starting prevalence reflected the assumed existing levels of interventions in addition to treatment, with no scale-up of these interventions after vaccine implementation. We assumed that treatment coverage for clinical cases was 40%. Computation time is roughly proportional to the number of infected individuals; therefore, at higher prevalence levels, we reduced the simulation size for computational efficiency. All simulations were repeated for 50 transmission model parameter sets, drawn from the posterior distribution of the model fit, to account for underlying parameter uncertainty.

### Profile exploration with three vaccine doses

We first considered the time-dependent antibody profile for a three-dose vaccine schedule, using the parameters described in White et al. [13]. We varied the parameters in the corresponding Hill function to systematically explore the impact of varying the initial efficacy (α) and duration of protection (β) on the transmission model outcome. The objective of this analysis was to determine the relative importance of initial vaccine efficacy versus duration of protection, and how this relationship changed across age groups and prevalence settings, while keeping the overall efficacy constant. We therefore calculated the area under the curve for the efficacy profile in Additional file 1: Figure S2, which replicates the efficacy profile fitted by White et al. using the results of the phase 3 RTS,S/AS01 clinical trial [4, 13]. We then obtained a set of α-β parameter pairs where the area under the curve was held constant, by fixing α and calculating the fitted β value using the Nelder-Mead optimisation method in R [16]. We used this method to create two additional efficacy curves: one for low initial efficacy and long duration, and another for high initial efficacy and short duration. Finally, we ran the transmission model for the range of efficacy profiles produced by these α-β pairs, for each transmission setting.

### Profile exploration with four vaccine doses

In the second analysis, we simulated the impact of a four-dose vaccine schedule administered to children aged 5–17 months. The results of the Regules et al. trial indicate that an enhanced RTS,S/AS01 vaccine could be obtained by varying dose timings and dosage amounts, and that efficacy can be improved without a significant increase in antibody concentration [6, 7]. In our vaccine model, antibody levels for the first three doses are fixed, and we vary the parameters of the function relating titre to efficacy to achieve different efficacy profiles. However, the fourth vaccine dose also assumes the same underlying relationship between titre and efficacy. Therefore, to determine the impact of varying efficacy following the fourth dose, we modified the titre at this dose to achieve different corresponding efficacy levels.

We first ran simulations for a range of values of the time interval between the third and fourth vaccine doses, and the antibody titre after the fourth dose. We then selected four titre-timing combinations for further analysis, and we derived the cumulative clinical cases averted in children younger than 5 years for these parameter pairs.

### Public health impact of childhood vaccination

Finally, we explored the potential public health impact of a hypothetical enhanced RTS,S/AS01 vaccine profile designed to correspond to the early clinical trial results in this area, compared to the results of the phase 3 trial [4, 6]. We created a set of three possible vaccine efficacy scenarios, with one scenario corresponding to the phase 3 trial data [4, 13]; another scenario approximating the phase 2a challenge study results [6, 7]; and an intermediate scenario. In these scenarios, the initial efficacy, duration, fourth dose titre and fourth dose timing were all varied (Table 2). We calculated the number of clinical cases averted across a range of prevalence settings and vaccine coverage levels (Table 1).

**Table 2:** Antibody model parameters

Scenario	*t*_dose 4_ (months)	*AB*_dose 4_ (EU/mL)	*V*_max_	α	β
A: RTS,S/AS01 phase 3	18	277	0.93	0.74	99.2
B: Intermediate	13	450	0.93	0.85	85.0
C: Modified	8	621	0.93	0.95	70.0

These parameters correspond to three vaccine scenarios: an RTS,S/AS01 phase 3 efficacy profile (A), a modified profile based on the phase 2a challenge study (C) [6, 7] and an intermediate profile (B). The antibody titre and efficacy profiles are provided in Additional file 1: Figure S6 and Fig. 4, respectively. The parameter ABdose 4 represents the antibody titre following the fourth vaccine dose at time tdose 4

### Sensitivity to outcome measure

For each simulation run we calculated the number of clinical and severe cases averted in 1-year age bands, per 1000 population per year, for 40 years following vaccine introduction. To measure vaccine impact, we calculated the number of cases averted as the difference in number of cases between the vaccine and baseline scenarios, per 1000 population. For the main results, we presented our findings in terms of clinical cases averted in 0–5-year-old children, in the first 10 years following vaccine introduction. This outcome was selected based on the reference to clinical malaria episodes in the World Health Organization Malaria Vaccine Technology Roadmap [3] and the time horizon and age cohorts over which a childhood malaria vaccine is most likely to be assessed. However, using the three-dose schedule efficacy profiles, we also tested the sensitivity of these outputs to a longer follow-up period, broader age range and to severe rather than clinical cases. For the analysis of the public health impact of a modified vaccine versus the current RTS,S/AS01 (with all four doses administered), we used three different measures to quantify vaccine impact: cumulative cases averted per 1000 0–5-year-old children; cumulative cases averted per 1000 fully vaccinated with four vaccine doses; and percentage reduction in cumulative cases in 0–5-year-old children.

## Results

### Profile exploration with three vaccine doses

**Fig. 2:**
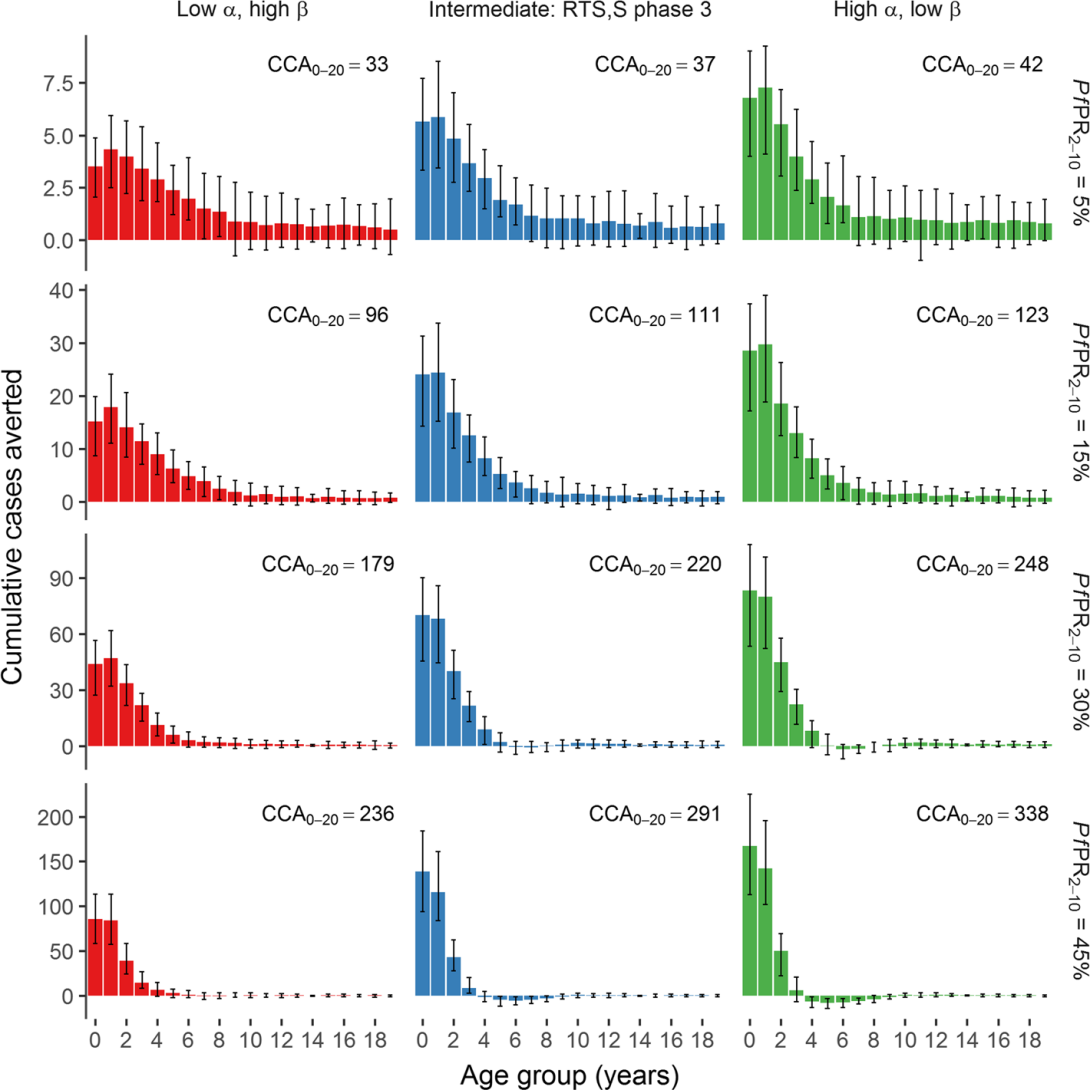
Population-level impact of the three-dose vaccine schedule. These figures show the total number of clinical cases averted over 10 years, for 1-year age groups up to 20 years of age. The total cases averted are shown per 1000 individuals in each age group, stratified by transmission setting (*rows*) and efficacy profile (*columns*) of Fig. 1. The *bars* show the median estimates, and the *error bars* show 95% credible intervals, based on 50 parameter draws. CCA_0–20_ represents the cumulative number of clinical cases averted over a 10-year period following the introduction of vaccination, in individuals younger than 20 years, per 1000 individuals. *Pf*PR_2–10_ represents *P. falciparum* prevalence for 2–10-year-old individuals, prior to vaccination

We derived three efficacy profiles where the overall efficacy (determined by the area under the efficacy-time curve) was held constant over a 10-year period following delivery of the third dose (Fig. 1). For the scenarios with higher initial efficacy, and in higher prevalence settings, the public health impact was more concentrated in younger age groups (Fig. 2). Further, the excess of cases in older children in the intervention group was more pronounced in these scenarios, compared to the lower transmission and longer duration scenarios, where the impact was more evenly spread across age cohorts. Overall the greatest number of clinical cases averted was obtained with a high initial efficacy, rather than a long duration of protection (Fig. 2). Additionally, we performed the same analysis with a fourth vaccine dose (Additional file 1: Figures S4 and S5) and for a wide range of α-β pairs (Additional file 1: Figure S3). In all transmission settings, the initial vaccine efficacy was the main determinant of the number of clinical cases averted.

### Profile exploration with four vaccine doses

**Fig. 3:**
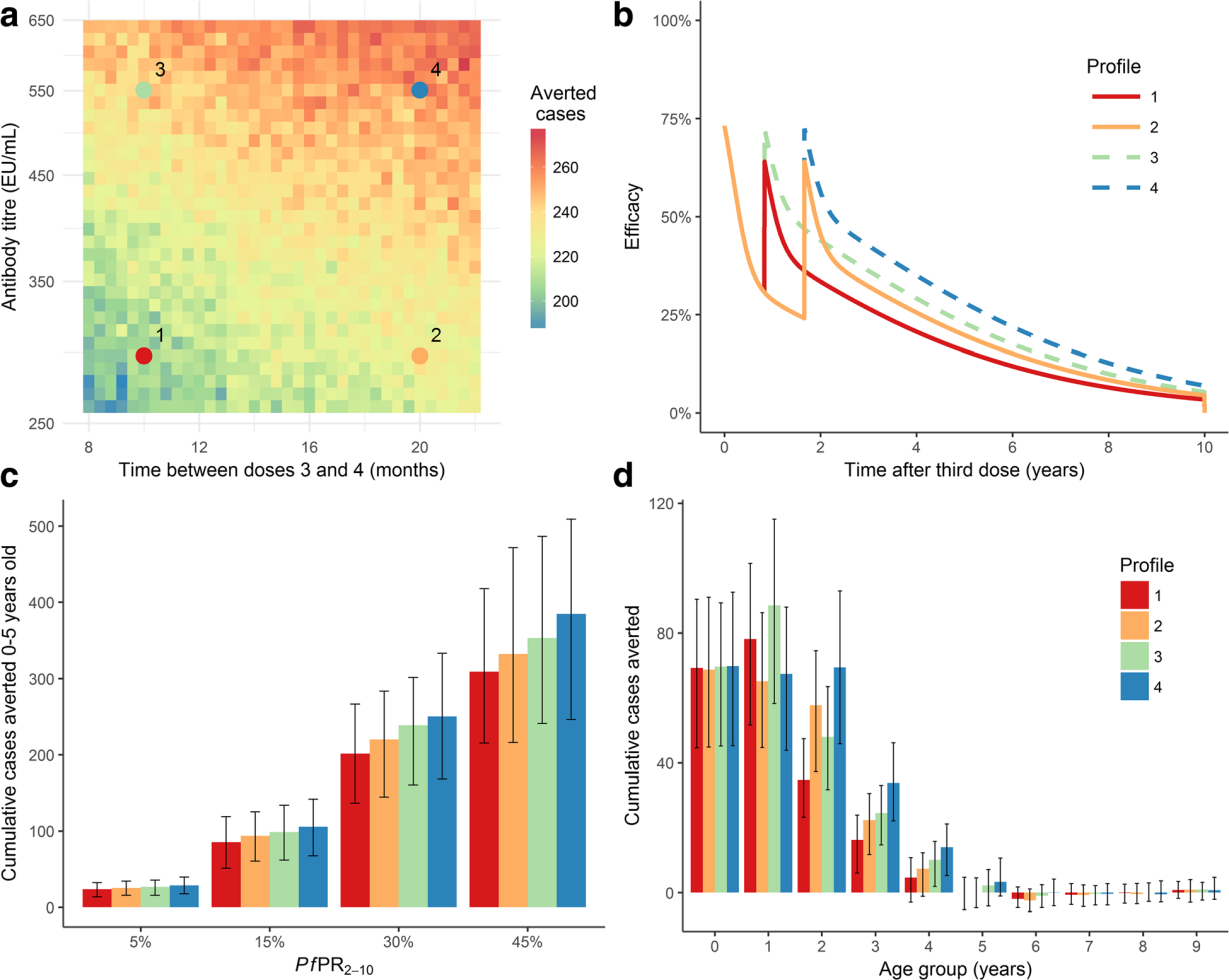
Impact of varying the timing and antibody titre of the fourth vaccine dose. **a** Cumulative clinical cases averted over a 10-year period in 0–5-year-old children, for a range of antibody titre and fourth dose timing values, where *Pf*PR_2–10_ = 30%. **b** Efficacy curves corresponding to the timing-titre parameter pairs indicated in panel **a**. **c** Cumulative clinical cases averted over a 10-year period in 0–5-year-old children, stratified by prevalence setting and efficacy profile. **d** Cumulative clinical cases averted over a 10-year period per 1000 individuals for 1-year age groups, where *Pf*PR_2–10_ = 30%. In **c** and **d**, the *bars* show the median estimates and the *error bars* show 95% credible intervals, based on 50 parameter draws. *Pf*PR_2–10_ represents *P. falciparum* prevalence for 2–10-year-old individuals, prior to vaccination

For the four-dose vaccine schedule, the greatest impact was achieved with a high antibody boosting at the fourth dose (which directly influenced efficacy) and a large interval between the third and fourth doses. Figure 3a shows the output for a transmission setting of *Pf*PR_2–10_ = 30%; however, the overall trend was consistent across all prevalence scenarios. In this part of the analysis, the area under the efficacy curve was not fixed, which resulted in the overall area under the curve of vaccine efficacy being higher when doses were spaced further apart, due to the primary series affording impact for a longer time. We found that the number of clinical cases averted correlated strongly with the overall area under the efficacy curve.

We selected four titre-timing combinations to create four efficacy scenarios: low efficacy and short timing; low efficacy and long timing; high efficacy and short timing; and high efficacy and long timing (Fig. 3a and b). The higher fourth dose efficacy and longer timing scenario resulted in the highest number of averted cases, and this effect was more marked in higher transmission settings (Fig. 3c). While the two higher efficacy (driven by the higher antibody titre) scenarios resulted in more averted cases overall, the longer timing schedules resulted in more averted cases in older age groups (Fig. 3d).

### Public health impact of childhood vaccination

**Fig. 4:**
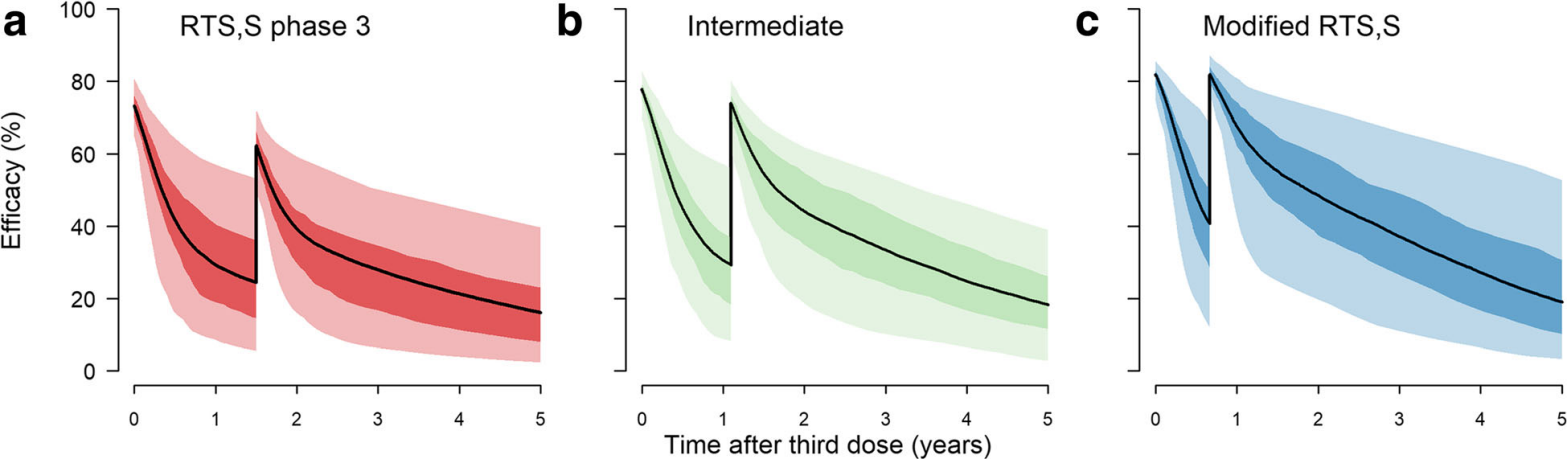
Time after the third vaccine dose (years), versus vaccine efficacy, for a four-dose schedule. Three scenarios are shown. **a** Fourth dose at 18 months, corresponding to the phase 3 trial data; **b** intermediate scenario; **c** fourth dose at 8 months with a higher antibody titre (corresponding to higher efficacy), approximating observed efficacy in the Regules et al. study, and referred to as the ‘modified’ vaccine scenario [6]. The parameters are given in Table 1 and the corresponding antibody titre curves in Additional file 1: Figure S6. In each subplot, the *solid line* is the median of 2000 simulations, and the *dark* and *light shaded* regions represent the 50% and 95% predictive intervals, respectively. Other parameters are those described in White et al, Table 3, for the 5–17-month age category [13]

Figure 4 shows the time-dependent vaccine efficacy over 10 years for the RTS,S/AS01 vaccine (Fig. 4a), a modified RTS,S/AS01 vaccine that approximately emulates the observations from the fractional third and fourth dose challenge study (Fig. 4c) and an intermediate efficacy scenario (Fig. 4b). The parameters used to create Fig. 4c were selected such that the efficacy following both the third and fourth doses was close to 90%, and the time between doses three and four was also reduced. To achieve a higher efficacy after dose four, we set the antibody titre following dose four to that observed after dose three in the phase 3 trial (i.e. 621 EU/mL). The parameters corresponding to the three scenarios are provided in Table 1, and the corresponding antibody titre plots are shown in Additional file 1: Figure S6.

**Fig. 5:**
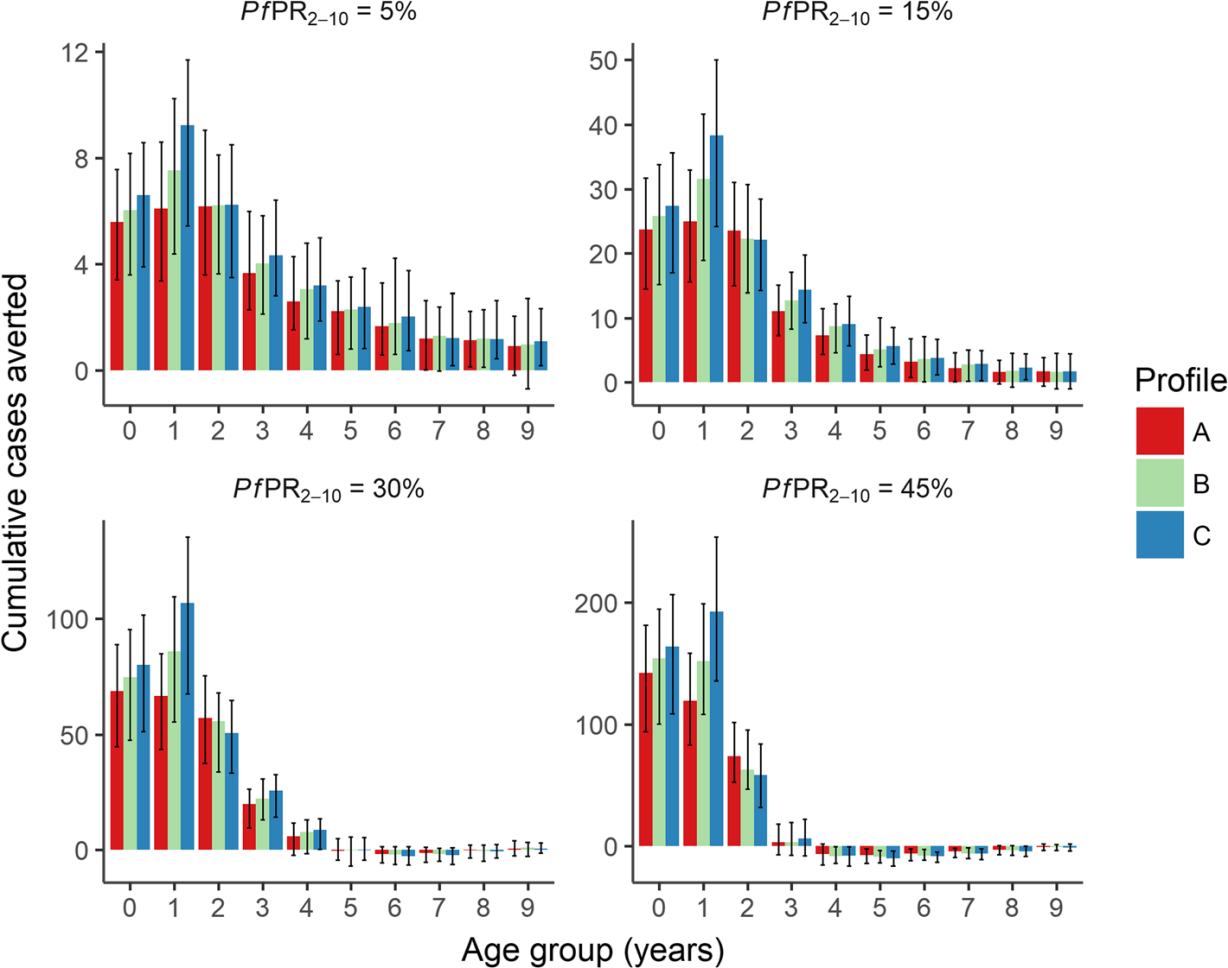
Population-level impact of the three efficacy scenarios in Fig. 4. Cumulative clinical cases averted over a 10-year period, per 1000 individuals, stratified by prevalence setting and efficacy profile. The *bars* show the median estimates, and the *error bars* show 95% credible intervals, based on 50 parameter draws. *Pf*PR_2–10_ represents *P. falciparum* prevalence for 2–10-year-old individuals, prior to vaccination

Comparing these three profiles, we found that the modified RTS,S/AS01 outperformed the profile based on the RTS,S/AS01 phase 3 data, even though the fourth vaccine dose was timed at 8 months, compared to 18 months (Fig. 5). In terms of cumulative cases averted per 1000 0–5-year-old children over a 10-year period, the modified vaccine schedule averted between 21 and 25% more clinical cases than the current RTS,S/AS01, across the four transmission settings (Additional file 1: Table S1). We found that in the lowest transmission setting where *Pf*PR_2–10_ = 5%, the RTS,S/AS01 phase 3 vaccine averted more cases in older children. We also observed a stronger shifting of cases from younger to older ages in higher transmission settings with the modified vaccine profile (Fig. 5). Cumulative clinical cases averted increased approximately linearly with higher vaccine coverage (Additional file 1: Figure S7).

### Sensitivity to outcome measure

We found that for the three-dose vaccine schedule analysis, measuring the cumulative clinical cases averted across all age groups, rather than children, and over a longer time horizon changed the results, with the low initial efficacy and long duration vaccine schedule becoming more advantageous at an earlier time point in low transmission settings (Additional file 1: Figure S8). However, where we used cumulative severe cases averted as the outcome measure, across all age groups there was almost no difference between the three efficacy profiles (Additional file 1: Figure S9).

For the analysis of the public health impact of three vaccine scenarios, where all four doses were administered, we included two additional outcome measures: clinical cases averted per 1000 fully vaccinated children, and percentage of clinical cases averted in 0–5-year-old children. The modified vaccine (scenario in Fig. 4c) was the most advantageous for all outcome measures, although the percentage reduction was higher in low malaria transmission settings.

## Discussion

In our study we used a dynamic modelling approach to compare the relative contributions of vaccine characteristics, to inform a TPP for a second-generation malaria vaccine for young children. We first examined the characteristics of initial efficacy and duration in the context of a three-dose schedule before analysing the characteristics specific to the fourth vaccine dose. Our results demonstrated that, for the three-dose vaccine schedule, initial efficacy was associated with a higher reduction in childhood clinical cases compared to vaccine duration. This effect was more pronounced in high malaria transmission settings due to the concentration of clinical cases in younger age groups, and this finding held true when a fourth vaccine dose was incorporated (where the fourth dose had the same efficacy and timing across all scenarios). However, we found that there was a larger age-shifting effect in higher transmission settings, where the initial efficacy was higher, due to the delayed acquisition of clinical immunity [17].

Examining the characteristics of the fourth dose, we found that the largest benefit in terms of clinical cases averted always occurred when the efficacy at the fourth dose was highest, and that a longer timing between doses three and four was also advantageous. In the third part of the analysis, we created three scenarios where all of the vaccine parameters — initial efficacy, duration and fourth dose titre and timing — were varied, to replicate the RTS,S/AS01 phase 3 profile; a profile approximately corresponding to the fractional dose study (referred to as the modified profile); and an intermediate profile. This analysis showed that a modified malaria vaccine would likely outperform the current RTS,S/AS01, but that increasing the timing between doses three and four could further reduce the number of clinical cases in older children.

The World Health Organization has specified that a malaria vaccine for children younger than 5 years should reduce incidence of all clinical malaria episodes by at least 75% for at least 1 year and preferably at least 2 years [3]. Importantly, the Preferred Product Characteristics state that initial efficacy and duration will be jointly considered, and the duration of protection is assessed as being as important as the short-term efficacy for the target vaccine group, in medium to high transmission settings [18]. Our findings indicate that, for imperfect vaccines with suboptimal efficacy, it may be advantageous to prioritise initial efficacy over duration.

The results of our study should be interpreted in the context of the selected follow-up time window, age cohorts and outcome measure. We focussed on children younger than 5 years and a 10-year horizon following vaccine introduction as our primary outcome measure, given the circumstances in which a childhood vaccine is likely to be assessed in field studies, as well as the many other changes to malaria interventions likely to occur over longer time periods. We found that the low initial efficacy and long duration profile resulted in more clinical cases averted when measuring cases in all age groups, and over a longer time window; however, this is likely due to the functional form of this efficacy profile allowing the vaccine benefit to extend for far longer than expected in practice. We also found that measuring the severe cases averted across all ages meant little difference in impact of the three efficacy profiles considered, further justifying the focus on childhood impact. Finally, our main outcome measure was clinical cases averted, and we did not account for the implementation costs associated with different schedules, which may change how vaccine efficacy and duration are prioritised.

Our study had several limitations. First, we fixed the age at first vaccine delivery at 5 months and did not incorporate any reduction in susceptibility to infection until after the third vaccine dose. It is possible that some protection is afforded after the first vaccine dose, which would further increase the vaccine impact. Additional data on the protection between doses would be needed to capture this in the modelling framework. Second, our proxy for total efficacy, i.e. total area under the time-efficacy curve over a 10-year period, meant that some of the area under the efficacy curve was outside the measured time window. However, our study focussed on impact in the childhood age group, and so we do not expect this assumption to greatly influence our results.

Our method assumes that there is a non-linear but positively increasing relationship between antibody titre and vaccine efficacy. This is biologically plausible based on findings from the RTS,S/AS01 vaccine. However, in the phase 2a challenge study there was not a great change in antibody levels between the protected and non-protected groups, suggesting that factors other than antibody titre may influence efficacy [6]. By varying the antibody titre at the fourth dose, we were able to capture the observed challenge study efficacy, without explicitly incorporating other factors into our antibody model, and these efficacy curves in turn informed the estimates of population-level vaccine impact. However, the efficacy of a second-generation vaccine may need to be improved by enhancing antibody quality or avidity, rather than by solely increasing antibody titre (given that higher titres may not be achievable in African children). Fitting of dose-response relationships to data from challenge studies for other malaria vaccines could be undertaken to incorporate other factors such as antibody quality into this modelling framework, to consider the potential public health impact of candidate vaccines in phase 2 studies.

Our analysis was motivated by the findings of the fractional third dose and fractional booster dose study by Regules et al. [6], which provides an early indication that changing dosage and timing could improve vaccine efficacy. However, the fractional dose study was conducted in a relatively small sample of 30 healthy malaria-naïve adults, meaning there are limitations in comparing these results with those from the phase 3 trial. In addition, it is not clear from this single study whether the increased efficacy is due to the fractional third dose or to the delay in the third dose [7]. A phase 2 trial of RTS,S/AS01E compared 0, 1, 2 month and 0, 2, 7 month dosing schedules within the Expanded Programme on Immunization. The trial found that over the 19 month study period, efficacy was higher in the 0, 1, 2 month group, although when comparing malaria episodes in the 12-month period following the third dose, efficacy across the two schedules was similar (noting that this study was in infants only) [19]. Further characterisation of the dose-response relationship between antibody titre and efficacy within this and future challenge studies could help elucidate this mechanism and guide TPP development. Furthermore, in creating the ‘intermediate’ vaccine efficacy profile, with parameters approximately at midpoints between those selected for the phase 3 and modified efficacy profiles, we implicitly assumed a trade-off between the timing of and antibody titre at the fourth dose, where a later dose timing resulted in a lower antibody titre (and therefore efficacy) following the fourth dose. A more complex relationship between these factors is possible and could be quantified given further data on this relationship.

Despite the success in developing the RTS,S/AS01 vaccine, there have been challenges, many of which will impact the progress of a second-generation vaccine. A key issue is that we still do not have a full understanding of the immunological mechanisms by which the RTS,S/AS01 confers protection against malaria disease, and there is no widely accepted immune correlate of protection. While there is evidence that high anti-CSP antibodies are associated with protection, antibody titre is not an established protective correlate. Further, it is likely that a second-generation vaccine will need to be assessed relative to the first-generation vaccine, which presents feasibility issues in terms of community acceptance, trial design and trial sample size. The measures by which the success of a new vaccine is assessed will also need to be carefully considered [20, 21].

## Conclusions

In vaccine development and evaluation, considerable focus is typically given to the durability, or half-life, of a vaccine. However, our findings show that these vaccine properties need to be considered in the context of the target population and immunological profile. In our study, we found that in the first decade of vaccine implementation the initial efficacy was more important than duration, based on our current understanding of the RTS,S/AS01 vaccine and in terms of childhood clinical cases averted. This study also emphasises the importance of considering the age distribution of incidence in a range of disease prevalence settings when predicting the impact of an age-targeted vaccine. Finally, our analysis demonstrates that the timing of the fourth RTS,S/AS01 dose can change the overall health benefit, which should be considered with the logistics of implementing a childhood malaria vaccine within the current Expanded Programme on Immunization framework. The findings from this analysis could provide insight for vaccine developers and policy-makers into how distinct properties of a malaria vaccine may translate to public health outcomes and how the importance of these characteristics changes across different malaria prevalence settings.

## Additional file

Additional file 1: Supplementary results. (DOCX 1310 kb)
