# Supplementary material for: Modelling population-level impact to inform target product profiles for childhood malaria vaccines
Source: BMC Med. 2018 Jul 13;16:109. doi: 10.1186/s12916-018-1095-6 (PMC6044028; doi:10.1186/s12916-018-1095-6)
Supplement: Supplementary file 1 — Supplementary results. (DOCX 1310 kb) [file 12916_2018_1095_MOESM1_ESM.docx]

**Additional file 1**

**
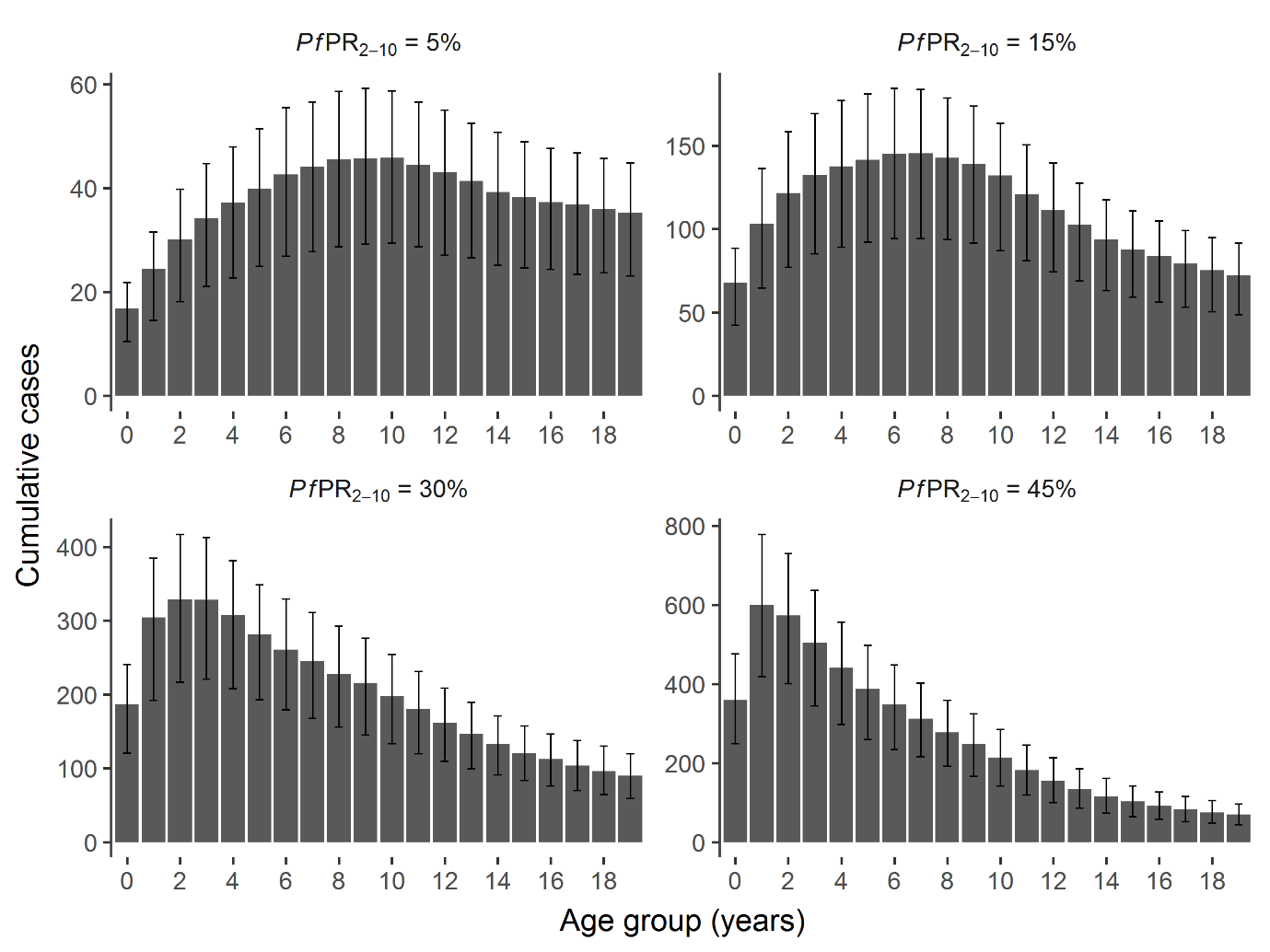
Figure S1. Baseline distribution of clinical malaria cases across age groups.** Cumulative clinical cases over a ten-year period per 1000 population, stratified by prevalence setting, in the absence of vaccination, produced by the individual-based malaria transmission model. This figure illustrates that in higher prevalence settings, malaria incidence shifts to younger age groups. *Pf*PR_2–10_ represents *P. falciparum* prevalence for 2–10-year-old individuals, prior to vaccination.


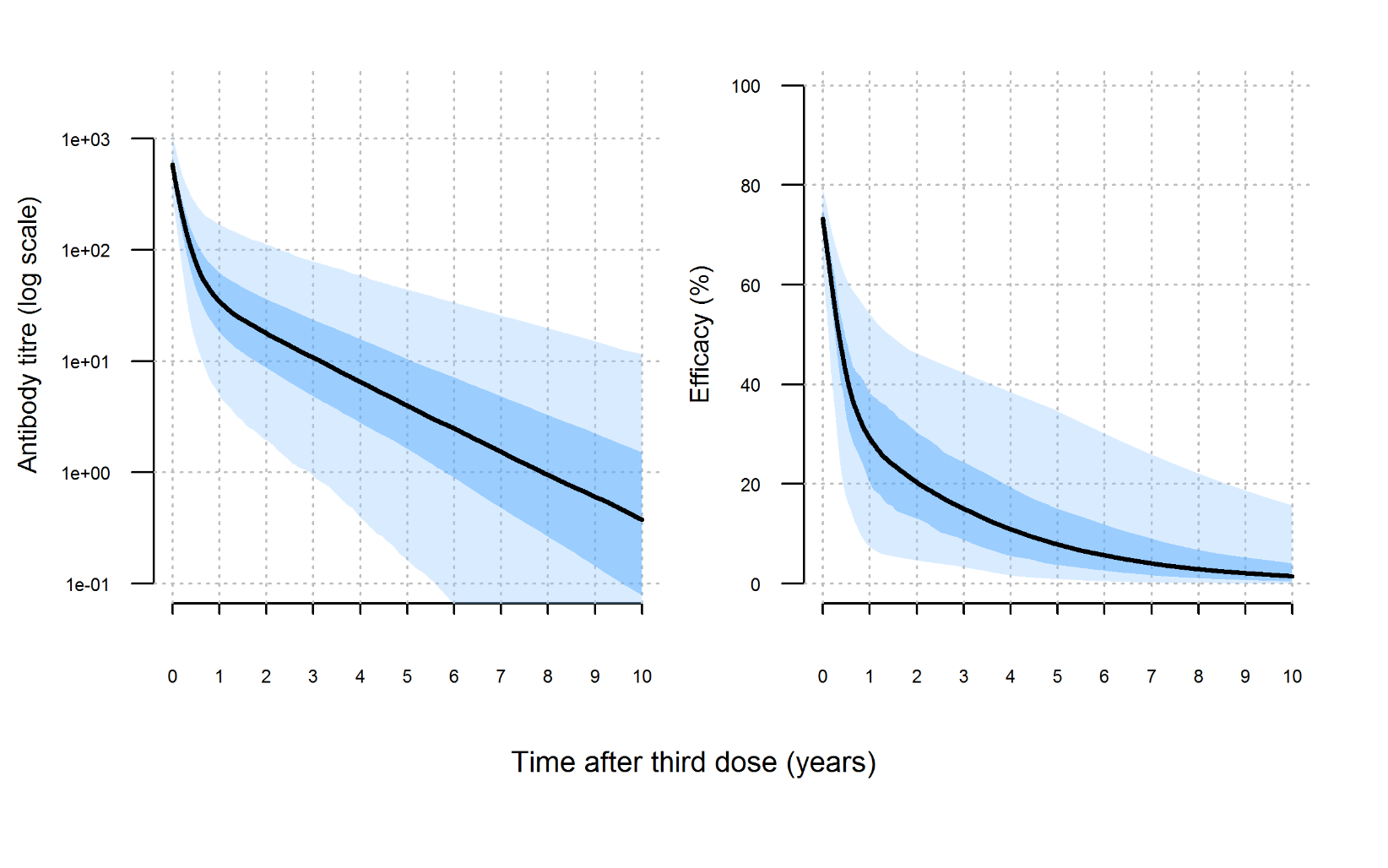


**Figure S2. Antibody titre and efficacy profile corresponding to the RTS,S/AS01 phase 3 trial, for a three-dose schedule**. Time after the third vaccine dose (years), versus the antibody titre. The heavy black line is the median of 2000 simulations, and the dark and light blue shaded regions represent the 50% and 95% predictive intervals respectively. The parameters are the fitted parameters described in White et al. (2015), Table 3, for the 5–17-month age category [1].


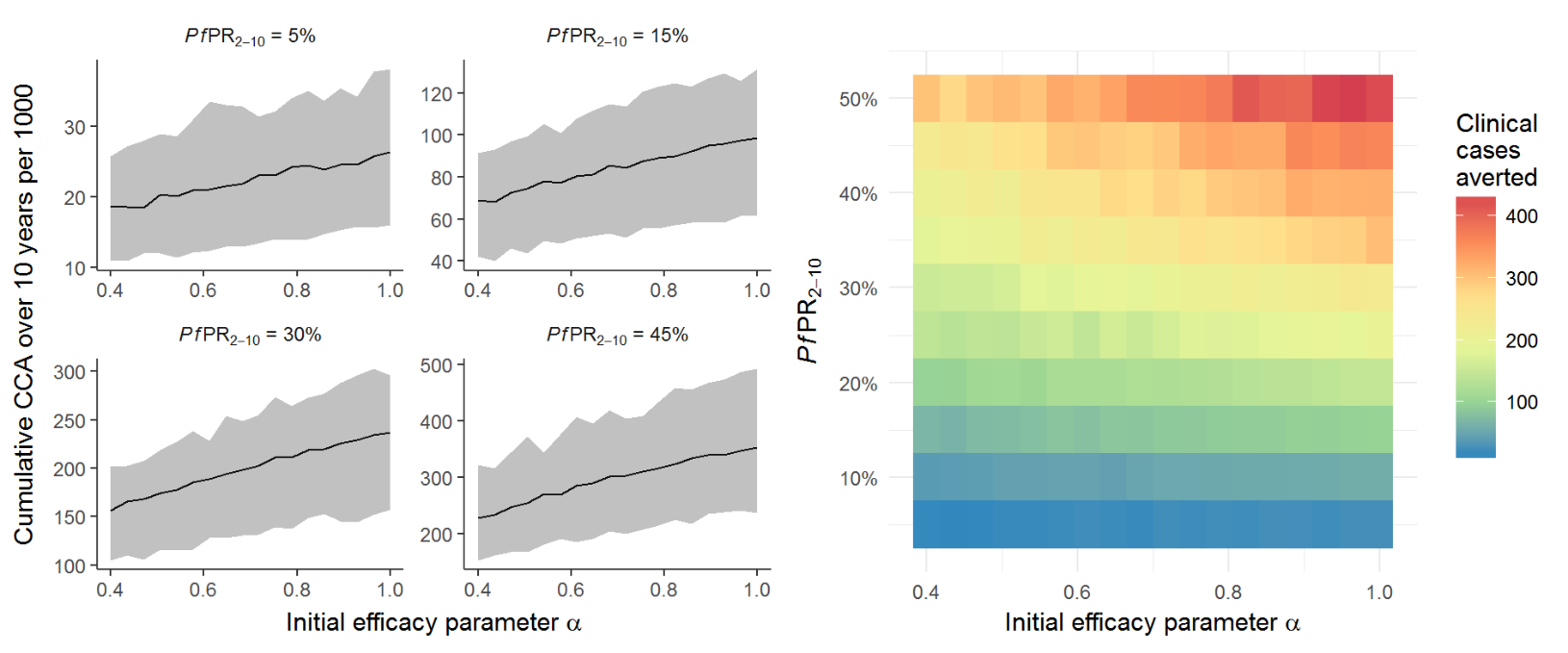


**Figure S3. Cumulative impact of the three-dose schedule.** Cumulative clinical cases averted (CCA) over a ten-year period following introduction of a three-dose vaccine schedule, in children younger than five years per 1000 population, for a range of values of the shape parameter α. The line plots show the number of averted cases for increasing α, for the four key transmission settings, where β is also varied such that the area under the efficacy curve is fixed. The heatmap depicts the number of averted cases across the range of α and prevalence settings.


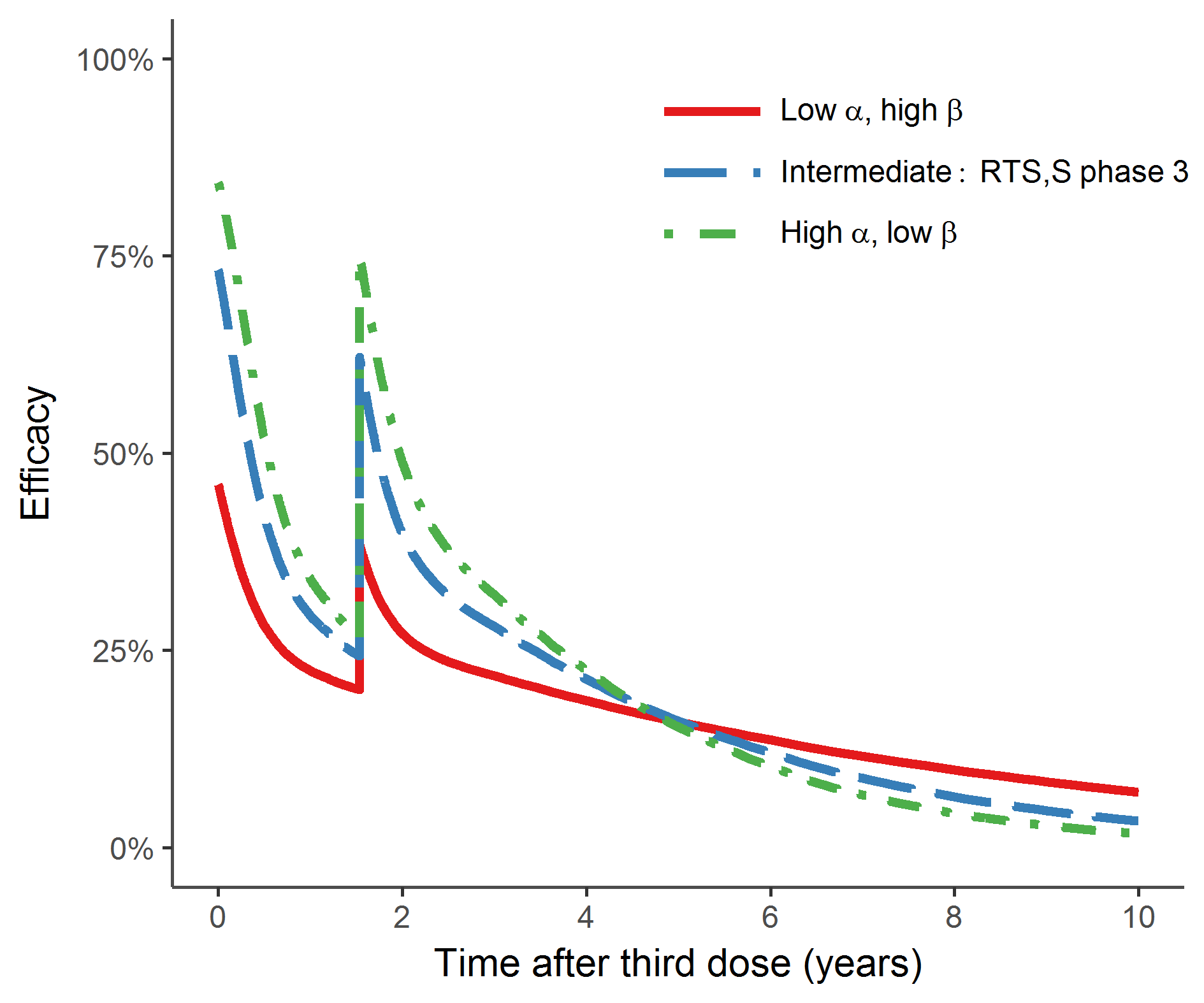


**Figure S4. Efficacy scenarios for a four-dose vaccine schedule, where fourth dose characteristics are unchanged.** Three vaccine efficacy scenarios where a fourth dose was incorporated, corresponding to the analysis in Figure S5. The fourth dose titre and timing parameters are the same for all three scenarios.

**
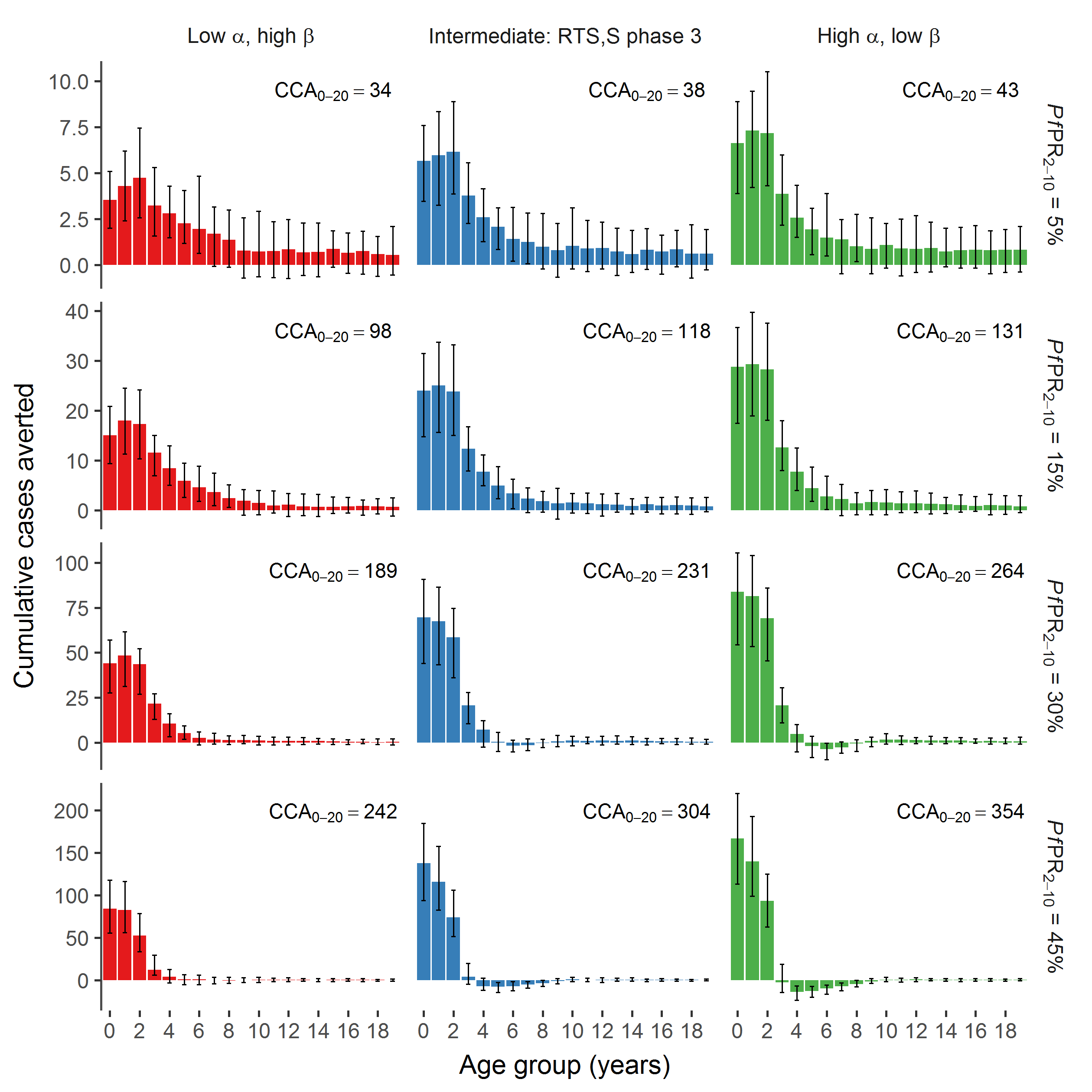
Figure S5. Population-level impact of the four-dose vaccine schedule**, **where fourth dose characteristics are unchanged.** Cumulative clinical cases averted a ten-year period, for one-year age groups up to 20 years of age. The total cases are shown per 1000 individuals in each age group, for the four transmission settings, and for the three efficacy profiles in Figure S4. The bars show the median estimates and the errors bars show 95% credible intervals, based on 50 parameter draws. CCA_0–20_ represents the cumulative clinical cases averted over a ten-year period following the introduction of vaccination, in individuals younger than 20 years, per 1000 individuals.

**
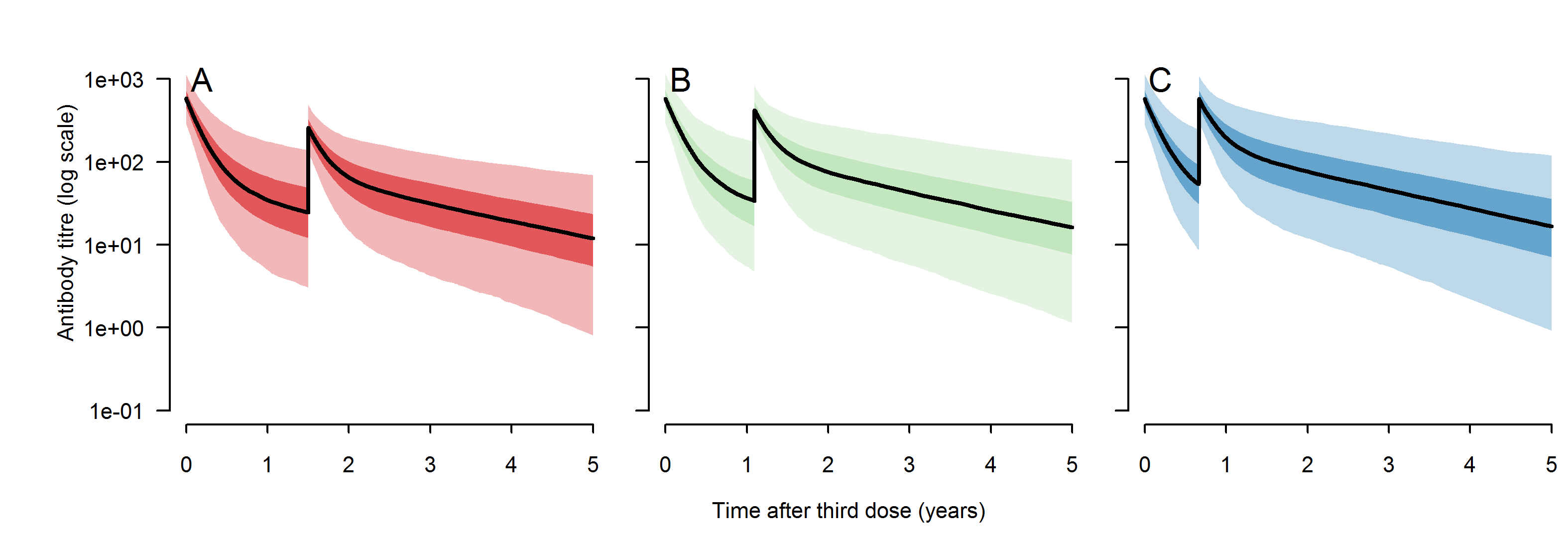
**

**Figure S6.** Time after the third vaccine dose (years) versus antibody titre, for a four-dose schedule. Three scenarios are shown. A) Fourth dose at 18 months, corresponding to the phase 3 trial data; B) Intermediate scenario; C) Fourth dose at 8 months with a higher antibody titre. The heavy black line is the median of 2000 simulations, and the dark and light shaded regions represent the 50% and 95% predictive intervals respectively. The parameters t_boost_ and AB_boost_ for each scenario are described in Table 1, and the corresponding efficacy profiles are in Figure 4. Other parameters are those described in White et al, Table 3, for the 5–17-month age category [1].


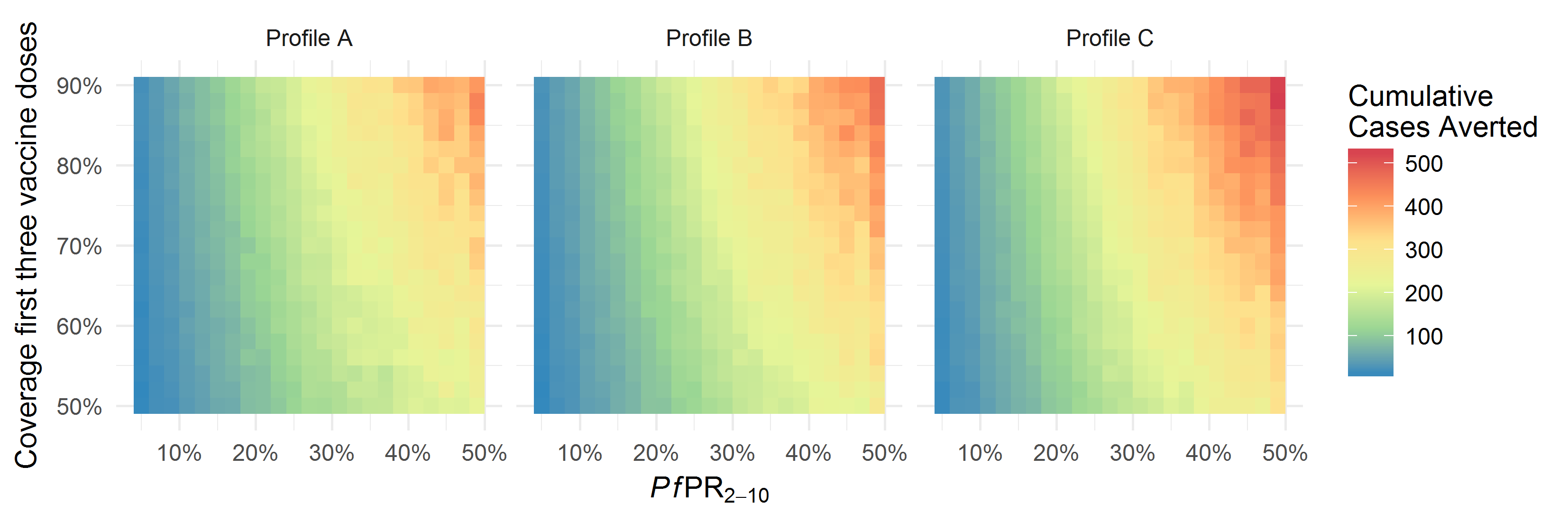


**Figure S7.** **Impact of vaccine coverage.** Cumulative clinical cases averted over a ten-year period following introduction of a four-dose vaccine schedule, in children younger than five years per 1000, stratified by profile and prevalence setting, for a range of vaccine coverage levels for the first three vaccine doses, where coverage of the fourth dose is set to 80% of that of the first three doses. The profiles A, B and C correspond to those in Figures 4 and 5.

**
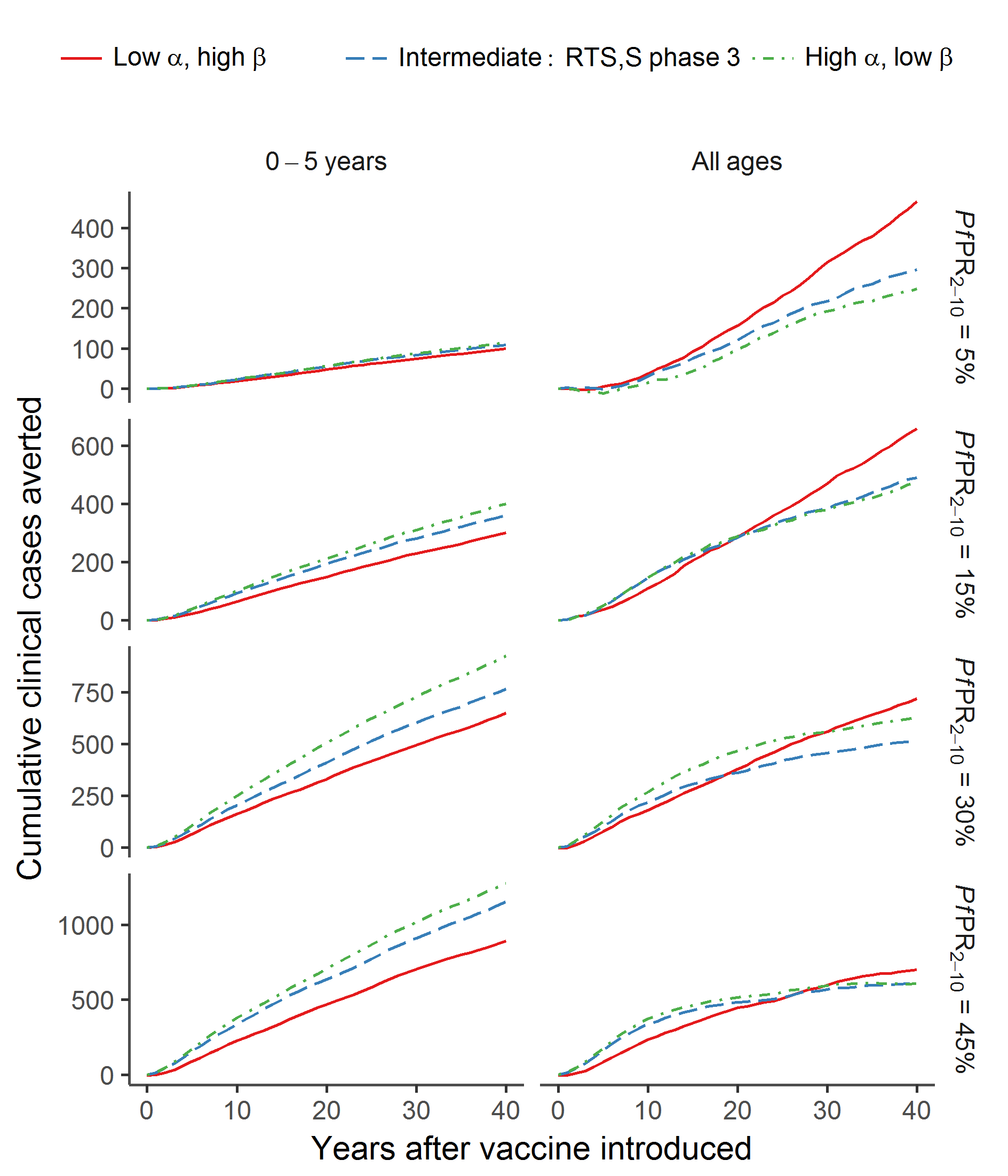
**

**Figure S8.** **Alternative outcome measure of longer time window.** Cumulative clinical cases averted per 1000 population over a 40-year period following vaccine introduction, for two age cohort groupings (0–5-year-old children, and all ages), and stratified by prevalence setting, for the three vaccine efficacy scenarios in Figure 1.


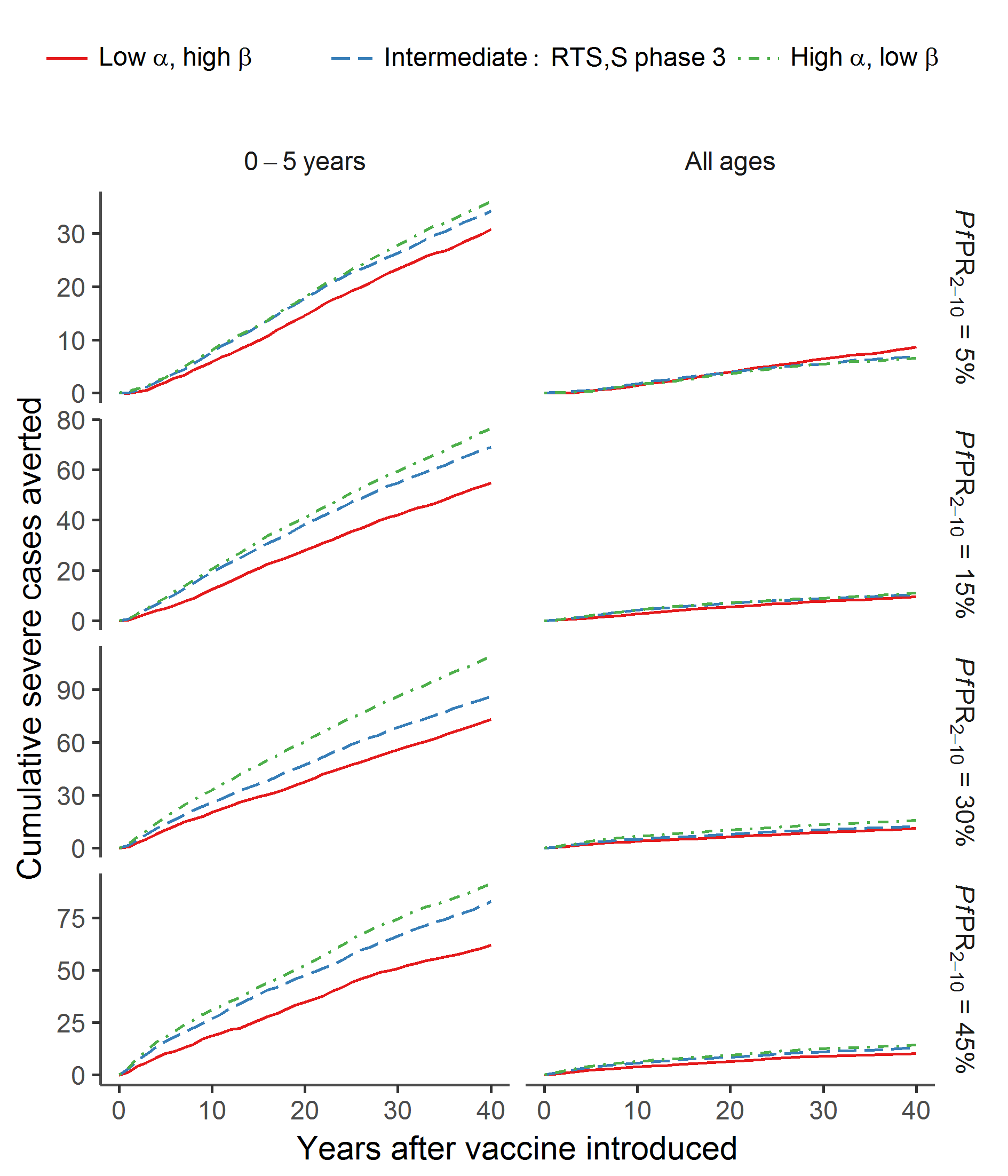


**Figure S9. Alternative outcome measure of severe cases and longer time window.** Cumulative severe cases averted per 1000 population over a 40-year period following vaccine introduction, for two age cohort groupings (0–5-year-old children, and all ages), and stratified by prevalence setting, for the three vaccine efficacy scenarios in Figure 1.


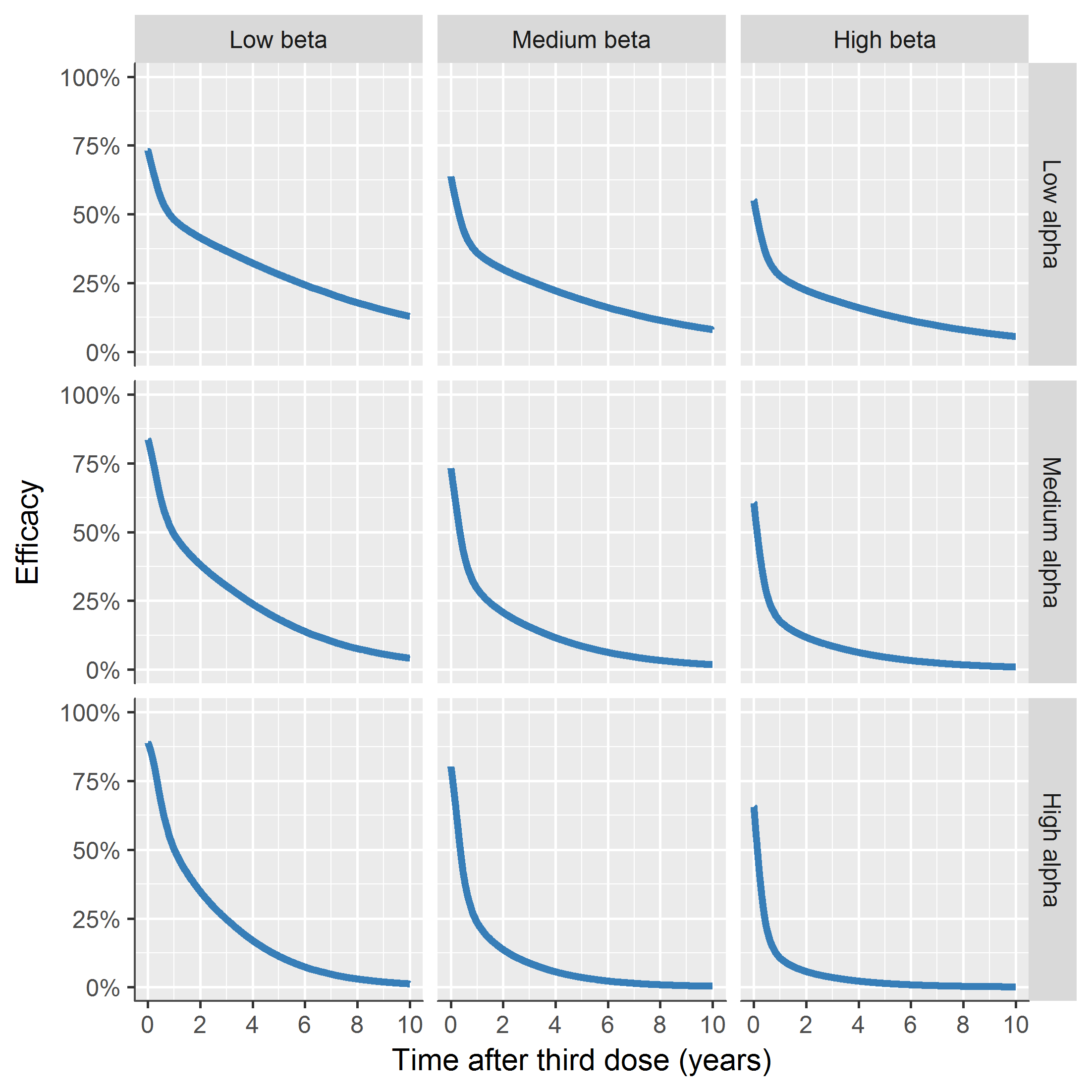


**Figure S10. Illustration of the impact of varying the parameters α and β in the vaccine efficacy model.** The equation for vaccine efficacy V(t) is $V\left( t \right)=V_{max}\left( 1-\frac{1}{1+\left( \frac{CS\left( t \right)}{\beta} \right)^{\alpha}} \right),$ where CS(t) is the antibody titre and V_max_ is the (fixed) maximum efficacy. Note that α is the shape parameter, and that increasing α increases the initial efficacy as well as the efficacy drop-off. The parameter β is the scale parameter, and Increasing β changes the magnitude of the efficacy over the entire time period and the slope of the efficacy curve. The shape and scale parameters can be adjusted to create efficacy profiles with high initial efficacy and a shorter duration (such as the lower left corner plot), or low initial efficacy and a longer duration (upper right corner plot).

**Table S1. Impact of a four-dose vaccine schedule for three efficacy scenarios.**

| **Prevalence setting and scenario** | **Clinical cases averted 0–5 years per 1,000 population** | **Clinical cases averted per 1,000 fully vaccinated** | **Percentage reduction in clinical cases in 0–5-year-old population (%)** |
| --- | --- | --- | --- |
| *Pf*PR_2-10_ *= 5%* | | | |
| A: RTS,S/AS01 phase 3 | 24.0 (14.9–33.5) | 292.2 (105.1–450.4) | 17.5 (15.4–19.2) |
| B: Intermediate | 27.2 (14.9–36.1) | 280.8 (111.6–487.0) | 19.1 (15.9–21.2) |
| C: Modified | 29.2 (17.7–38.8) | 316.5 (178.8–456.2) | 20.8 (19.0–23.5) |
| *Pf*PR_2-10_ *= 15%* | | | |
| A: RTS,S/AS01 phase 3 | 92.8 (57.7–120.4) | 730.3 (449.7–1006.3) | 16.4 (15.4–17.7) |
| B: Intermediate | 101.2 (61.6–135.5) | 767.1 (490.8–1059.8) | 18.1 (16.8–19.2) |
| C: Modified | 113.0 (71.0–145.8) | 802.4 (513.0–1015.1) | 19.8 (18.8–21.0) |
| *Pf*PR_2-10_ *= 30%* | | | |
| A: RTS,S/AS01 phase 3 | 213.2 (146.0–276.6) | 1290.9 (909.3–1692.4) | 15.2 (13.9–16.2) |
| B: Intermediate | 244.3 (156.9–302.7) | 1402.2 (915.9–1711.8) | 16.8 (15.6–17.8) |
| C: Modified | 267.4 (176.3–334.4) | 1386.3 (964.5–1820.3) | 18.6 (17.3–19.5) |
| *Pf*PR_2-10_ *= 45%* | | | |
| A: RTS,S/AS01 phase 3 | 334.9 (223.6–431.9) | 1781.2 (1153.7–2401.8) | 13.4 (12.1–14.9) |
| B: Intermediate | 365.8 (251.3–503.5) | 1846.3 (1223.9–2586.8) | 14.8 (13.5–16.3) |
| C: Modified | 413.3 (284.0–539.3) | 1983.6 (1344.4–2563.2) | 16.7 (15.4–17.9) |

Median estimates and 95% credible intervals, based on 50 parameter draws, of the cumulative clinical cases averted over a ten-year period following introduction of a four-dose schedule vaccine. Efficacy scenario A corresponds to the RTS,S/AS01 vaccine as observed in the phase 3 trial [1], scenario C approximates the findings from the phase 2a challenge study [2], and scenario B represents an intermediate efficacy profile. The parameters for the three scenarios are in Table 2 in the main manuscript, and the efficacy profiles and modelled outcomes are in Figures 4 and 5 in the main manuscript.

**References**

1. White MT, Verity R, Griffin JT, Asante KP, Owusu-Agyei S, Greenwood B, et al. Immunogenicity of the RTS,S/AS01 malaria vaccine and implications for duration of vaccine efficacy: Secondary analysis of data from a phase 3 randomised controlled trial. Lancet Infect Dis. 2015;15:1450–8.

2. Regules JA, Cicatelli SB, Bennett JW, Paolino KM, Twomey PS, Moon JE, et al. Fractional third and fourth dose of RTS,S/AS01 malaria candidate vaccine: A phase 2a controlled human malaria parasite infection and immunogenicity study. J Infect Dis. 2016;214:762–71.
